# Refilling Prescriptions

**Published:** 1911-11

**Authors:** 


### Refilling Prescriptions

THE National Association of Retail Druggists, at its meeting at Niagara Falls in September, 1911, discussed this subject at length and finally decided to honor a written order not to refill a prescription but to pay no attention to instructions to this effect, printed upon a prescription blank.

In our opinion, the only thoroughly satisfactory conception of a prescription is in analogy with a bank check; namely, that it is an order to pass out to a certain individual or his authorized representative, a definite amount at a given time. We do not see why a patient should keep a prescription and have it refilled whenever he chooses or pass it on to his friends, in the original or by copy, and have them collect it, whenever they choose, any more than a check should be similarly treated. It certainly is convenient—and cheap—to refill a prescription indefinitely. It would be exceedingly convenient if we could use checks in the same way. Nor do we see why it should be necessary to mark a prescription not to be repeated, any more than a check.

However, it is some satisfaction to know that some definite action has been taken by the pharmaceutic profession and, after all, it often is convenient to order a prescription repeated verbally. And we must not forget that, from the business standpoint, the free access of the laity to drugs, singly or combined, does the physician about as much harm as the artist would suffer if the public could get hold of his paints and brushes and thus compete with the professional painter.

